# Housing and health inequalities: A synthesis of systematic reviews of interventions aimed at different pathways linking housing and health

**DOI:** 10.1016/j.healthplace.2010.09.011

**Published:** 2011-01

**Authors:** Marcia Gibson, Mark Petticrew, Clare Bambra, Amanda J. Sowden, Kath E. Wright, Margaret Whitehead

**Affiliations:** a MRC Social and Public Health Sciences Unit, Glasgow, UK; b Public and Environmental Health Research Unit, Department of Public Health and Policy, London School of Hygiene and Tropical Medicine ,1 Keppel St., London WC1E 7HT, UK; c Department of Geography, Durham University, Wolfson Research Institute, Durham University Queen's Campus, Stockton on Tees TS17 6BH, UK; d Centre for Reviews and Dissemination, University of York, York YO10 5DD, UK; e Division of Public Health, University of Liverpool, Whelan Building, Quadrangle, Brownlow Hill, Liverpool L69 3GB, UK

**Keywords:** Housing, Health inequalities, Systematic review, Intervention, Evidence

## Abstract

Housing and neighbourhood conditions are widely acknowledged to be important social determinants of health, through three main pathways: (1) internal housing conditions, (2) area characteristics and (3) housing tenure. We conducted a systematic overview of systematic reviews of intervention studies to provide an overview of the evidence on the impact of housing and neighbourhood interventions on health and health inequalities. There is relatively strong evidence for interventions aimed at improving area characteristics and compelling evidence for warmth and energy efficiency interventions targeted at vulnerable individuals. However, the health impacts of area-level internal housing improvement interventions are as yet unclear. We found no reviews of interventions aimed at altering housing tenure. This remains an important area for further research and potentially new evidence syntheses.

## 1 Introduction

Health inequalities persist in developed countries, despite general improvements in health outcomes across the population (Department of Health, 2008). The unequal distribution of health has led to a growing awareness that health is socially determined by factors originating in different levels of society, ranging from the individual to the structural, as represented by models such as Dahlgren and Whitehead’s well-known ‘rainbow’ model (Dahlgren and Whitehead, 2007). At the individual level, biological and behavioural factors influence health, and are amenable to healthcare or behavioural interventions—these are known as ‘downstream’ interventions because they are located closest to the apparent source of health problems. At the other end of the spectrum, structural factors such as employment conditions and education also influence health, and because they are unevenly distributed, play a role in the creation and maintenance of health inequalities. Interventions aimed at these ‘upstream determinants’ are therefore required to tackle health inequalities (Acheson, 1998; Graham, 2004). Housing and neighbourhood conditions are such an upstream determinant. Thus, attempts to improve housing and neighbourhood conditions are seen as upstream interventions with the potential to tackle health inequalities, the importance of which was recently highlighted by the WHO report of the Commission for Social Determinants of Health (2008).

Factors linked to housing and neighbourhood conditions that influence health can be grouped into three broad categories, or pathways (Acevedo-Garcia et al., 2004): area characteristics, internal housing conditions and housing tenure, all of which have been shown to have independent effects on health (Shaw, 2004). Within each of these pathways, there are numerous specific mechanisms, which may influence health. Area characteristics may impact on health in many ways. Deprived areas may experience higher levels of crime and social disorder, making it more stressful and dangerous to live in them. Access to amenities may be worse than in more affluent areas, and there may be fewer jobs available. Cultures influencing health behaviours and employment may differ, and both residents and outsiders may perceive neighbourhoods negatively (Kling et al., 2007). Many aspects of internal housing conditions have the potential to influence health. In particular, cold and damp conditions may cause or exacerbate respiratory health conditions. Poisoning may be caused by lead piping, lead paint or carbon monoxide, and injuries may be caused by accidents related to high-rise housing or lack of safety equipment, particularly among children and the elderly. Lack of smoke alarms, fire extinguishers and sprinklers may exacerbate the risk of injury from fire (Shaw, 2004). There is some evidence that housing design and street layout can impact on psychosocial outcomes (Gibson et al., in press). Housing tenure may have psychosocial impacts on health; owning one’s one home may confer greater feelings of security or prestige than social or private renting, and is often used as an indicator of greater long-term command over resources. Conversely, the burden of debt involved may lead to anxiety and worry. Renting a home may be seen as socially inferior in some national or historical contexts but not in others (Macintyre et al., 2003). It is difficult to disentangle the impacts of these pathways; for instance, it would appear that much of the relationship between housing tenure and health is explained by the worse internal housing conditions and greater level of area problems associated with social housing (Macintyre et al., 2003). There is therefore a need to identify and collate the evidence on interventions, which aim to improve health by addressing each of these multi-factorial pathways linking housing to health: (1) area characteristics, (2) internal housing conditions and (3) housing tenure.

The use of systematic reviews to locate, appraise and synthesise evidence in fields such as housing and public health has become increasingly common, as the growing number of studies on given topics necessitate a thorough overview to make these studies accessible and to contextualise conflicting findings. However, the recent proliferation of systematic reviews has been such that it has now become difficult to keep pace with their output (Petticrew et al., 2008). Thus, so-called meta- or ‘umbrella’ reviews have emerged as a means of identifying, appraising and synthesising the evidence contained in systematic reviews and of making findings accessible to policy-makers and practitioners (Bialy et al., 2006; Egan et al., 2008). Presenting the overarching findings of reviews of ‘upstream’ interventions is arguably increasingly important given the growing recognition of the importance of structural factors in the social patterning of health. For these reasons, we conducted a systematic overview of systematic reviews of housing and community interventions with three main aims:

(1) to identify what types of housing and neighbourhood interventions have been reviewed systematically and how these relate to the different pathways between housing and health;
(2) to establish what gaps exist in the systematic review evidence base on housing interventions; and
(3) to consider what existing reviews can tell us about the impact of housing and neighbourhood interventions on health and health inequalities.

## 2 Methods

Systematic review methods were used to locate and evaluate published and unpublished systematic reviews of studies that evaluated the impact of housing and community interventions on health and health inequalities. We sought reviews of housing interventions, which focused on **health outcomes**, and particularly on health inequalities, whether via the targeting of interventions at disadvantaged groups or by reporting differential impacts according to social sub-groups.

### 2.1 Search strategy

The searches were designed and conducted by an experienced Information Scientist from the Centre for Reviews and Dissemination (CRD). We searched the CRD Wider Public Health database manually from 2000–2002. In addition, we conducted electronic searches of the Cochrane Database of Systematic Reviews, the Criminal Justice Abstracts database (2000–2007) and the Database of Abstracts of Reviews of Effects (DARE; 2002–2007), and hand searched the Campbell Collaboration Database and the Evidence for Policy and Practice Information and Coordinating Centre database (2002–2007). (An example of the search strategy used to use search the DARE database can be viewed at Appendix A.) Bibliographies and relevant websites were searched, and experts were contacted. Four journals were hand searched: American Journal of Public Health, American Journal of Preventive Medicine, Journal of Epidemiology and Community Health, Social Science and Medicine (2002–2007). Through our own contacts, we were also aware of one review (Thomson et al., 2009) conducted outwith the search time frame, which we included because it represents a major contribution to the evidence base on internal housing conditions and health.

### 2.2 Inclusion and exclusion criteria

Only reviews from 2000–2007 of adult participants (16+) or the general population in OECD countries (North America, Europe, Australasia, Japan) were eligible for inclusion. Reviews of studies evaluating interventions aimed at altering housing or neighbourhood conditions, which collected data on health or well-being outcomes were included. Systematic reviews had to meet what were then the two mandatory criteria of the Database of Abstracts of Reviews of Effects; that there is a defined review question and that an effort has been made to identify all the relevant literature. A minimum of one or more named databases needed to be searched, in conjunction with either reference checking, hand-searching, citation searching or contact with authors in the field to meet the second criterion.

### 2.3 Data extraction and critical appraisal

**Fig. 1:**
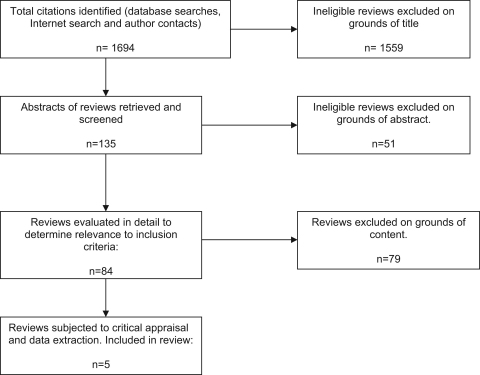
Flow diagram of included reviews.

All titles and abstracts (*n*=1694) were independently screened by two reviewers (MG/CB), and relevant reviews (*n*=84) were retrieved and assessed for inclusion. The flowchart is provided in Fig. 1. Discrepancies were resolved by consensus, or referred to a third reviewer if necessary (MP). Data relating to the review methods (search strategy, inclusion criteria, synthesis) were extracted along with information about the intervention, participants, outcomes, results (including number of studies and study design), authors’ conclusions and research recommendations. Each systematic review was critically appraised using a checklist list adapted from DARE. The checklist was used to highlight variations in the reviews and to assess their reliability and validity (see Box 1).

## 3 Results

Five reviews met the criteria for inclusion, containing a total of 130 studies of relevance to this overview of reviews, although the number of unique studies is smaller, as there is some overlap between the reviews. Three of the systematic reviews we included were aimed at addressing pathway one—the impact of area characteristics. Two of these were reviews of US interventions aimed at reducing the concentration of poverty by using various means to relocate families living in high poverty areas to more affluent areas (Acevedo-Garcia et al., 2004; Anderson et al., 2003). The third assessed area-based urban regeneration interventions in the UK (Thomson et al., 2006). One review addressed pathway two, internal housing conditions and included studies of warmth and energy efficiency interventions, housing improvement, refurbishment and relocation (Thomson et al., 2009). The fifth review included a range of housing interventions aimed at several of the pathways linking housing to health, including rehousing, injury prevention and behaviour change interventions (Saegert et al., 2003). No reviews relating to the third pathway, altering housing tenure, were located. The results are summarised in Tables 1–3, and a narrative synthesis is presented below. We also relate these findings to a recent literature review from the WHO Regional Office for Europe, which summarised the evidence of the extent of social inequities in risk associated with housing and residential location (Braubach and Fairburn, 2010). This review considered risks derived from seven sources: (i) housing and indoor environments, (ii) fuel poverty and thermal comfort, (iii) indoor environmental exposures and overcrowding, (iv) water and sanitation, (v) outdoor environments and residential location, (v) neighbourhood deprivation, safety and physical activity (vi) noise and (vii) pollution and environmental deprivation. This allows us to consider whether the systematic reviews we uncovered (which assess the effectiveness of interventions) tell us how to intervene within the main risk categories identified in the Braubach and Fairburn (2010) review.

### 3.1 Pathway 1: reviews of interventions aimed at improving area characteristics

#### 3.1.1 Tenant based rental assistance programmes

Tenant based rental assistance programmes aim to influence area characteristics by moving disadvantaged people from areas of high poverty to areas of low poverty. We found two reviews, which considered the health impacts of these kinds of intervention. It should be noted though that in many cases the primary aim of the interventions was economic, that is to increase labour market activity and economic self-sufficiency.

A US review of interventions designed to address area characteristics (Anderson et al., 2003) searched for evaluations of mixed income housing developments in areas of high poverty, but found none of sufficient quality for inclusion. They also searched for interventions providing low-income families with housing vouchers to allow them to move to more affluent areas. Twelve studies were located evaluating four such programmes: the Housing Allowance Experiment, Housing and Urban Development (HUD), Section 8 and Gautreaux and Moving to Opportunity (MTO). They included randomised controlled trials (RCTs; the MTO studies) and controlled and uncontrolled prospective studies. The programmes subsidised housing costs for families with income below 50% of the area median, allowing them to seek accommodation in the private rental market. Intervention group participants were required to move to a lower poverty area and to remain in that area for a minimum of a year post-intervention. The families then contributed 30% of their monthly income to the rental costs, with the remainder being subsidised as part of the intervention. The authors analysed outcome data by summing similar outcome measures within and between studies, then calculating a median effect across all of the outcomes. From these measures, it was not possible to deduce anything about which study reported the measure, the study quality, sample size or whether any studies reported adverse effects. Data were presented for each outcome measure in the Appendix. Thirty measures of crime and social disorder across 10 studies were reported. The majority of these improved significantly in favour of the intervention group. A significant decrease in four health and safety risks was found by one study (MTO) of lower quality (range 28–53% in rodent infestation, peeling paint, inadequate plumbing and broken or missing locks). In two good quality studies (Boston and New York MTO), significant improvements in 14 measures of adult mental health were found, and there were significant improvements in two measures of self-reported health (11% increase in self-reported good or excellent health in New York, effect size 32%, *p*=0.1 and 12% increase in Boston, effect size 20% *p*=0.05). The authors conclude that there is insufficient evidence on physical and mental health outcomes on which to base any policy recommendations. However, they recommend such interventions as a means to improve household safety. This review met six out of seven critical appraisal criteria; it was unclear whether more than one author had been involved at each stage of the review process. The publications in the review were quality appraised, but the appraisal tool was not provided, and the manner of describing individual study quality did not permit identification of each study’s quality, or of the numbers attaining a given level of quality. The role of study quality in interpreting the findings of the review was not discussed.

Another US review (Acevedo-Garcia et al., 2004) included evaluations of US housing policies aimed at addressing area characteristics, which included at least one health outcome (mental or physical health, experience of violence, health behaviours and medical care). Included studies had to have a comparison group. This review examined a wider range of housing mobility policies with a closer focus on health outcomes. They located 13 such studies, of five housing mobility programmes. Three of these (Gautreaux, Section 8 [this reported only child outcomes and is not reported here], and MTO) used various combinations of Section 8 vouchers alone (to allow families to move to private rented accommodation in more affluent areas), or Section 8 vouchers plus counselling. Two further ‘scattered-site’ interventions (Yonkers and Cincinnati) involved building new public housing units in low poverty areas.

Quasi-experimental studies of the Gautreaux programme found that tenants who moved within the city reported greater satisfaction with medical care than at baseline, while those who moved to the suburbs reported lower satisfaction than did city movers. The MTO programme (all studies were RCTs) was delivered in five US metropolitan sites, and included treatment (Section 8 voucher limited to low poverty area plus counselling), Section 8 (unrestricted voucher without counselling), and control (no voucher, but remain eligible for public housing) groups. After 2–3 years, the Boston MTO reported a small but significant decrease in personal crime victimisation, and significant increases in both overall health and ‘feeling calm and peaceful’ for both treatment and Section 8 groups. The New York MTO treatment group reported significant decreases in distress symptoms and exposure to violence. There were also indications of positive health and social outcomes for children and adolescents across all the sites, although these were mediated by gender. An interim report published in 2003 (on all of the MTO study sites 4–7 years post-intervention) did not report site-specific results, but found that there were large and significant reductions in obesity for the treatment and Section 8 groups, and in mental health problems for the treatment group. No differences in outcomes by ethnicity, socio-economic status (SES) or gender were reported. Of the ‘scattered-site’ interventions, one Cincinnati study reported a significant reduction in personal attacks and robberies, and an increase in employer provided healthcare benefits, while two Yonkers studies found that those who relocated reported significantly lower depression symptoms, problem drinking, marijuana use and experience of violent or traumatic events. No data on effect sizes or sample sizes were reported.

The review authors conclude that both tenant- and unit-based residential mobility programmes have the potential to improve health and health behaviours, but since there are few experimental or quasi-experimental studies, more research is needed to confirm this. They also note the methodological limitations of many of the studies, notably the risk of selection bias even in the randomised studies; there is some evidence to suggest that individuals with existing health problems were more likely to remain in their area of origin. The authors also point out that changes in internal housing conditions, rather than area characteristics, may account for improvements in health outcomes for those who moved. They argue that hypothesised causal pathways need to be elucidated more clearly and tested more rigorously via collection of qualitative data, data on internal housing conditions, and more detailed neighbourhood data including validated measures of neighbourhood quality. This review met all of the critical appraisal criteria. The included studies were quality assessed, and a table was provided, which allowed clear identification of each study’s quality. The full quality appraisal tool was not provided, but a reference to the tool used was provided, with some explanation of the parameters for assessing quality. In addition, the authors discussed potential threats to validity in each study and considered how these influenced interpretation of the evidence as a whole.

#### 3.1.2 Urban regeneration

The health and social impacts of UK urban regeneration programmes, or area-based initiatives (ABIs), which aim to tackle area characteristics by improving deprived areas, were the focus of a review by Thomson et al. (2006). Such programmes are aimed at the entire population of the target area, which are often mixed in their socio-economic composition; thus, those affected by the intervention are not necessarily personally disadvantaged. Three uncontrolled evaluations of ABIs reported data on health outcomes: two evaluated the Single Regeneration Budget (SRB), a multi-agency ABI aimed at improving employment, training, economic growth, housing, crime, environment and quality of life; and one evaluated New Life for Urban Scotland, which was also a multi-agency ABI targeted at housing, the environment, service provision, training and employment. One panel study of the SRB found that three out of four measures of self-reported health got worse, by up to 3.8% over three years. Two evaluations of case study areas using routine data found that standardised mortality rates improved (131 v 114 in the New Life programme, 122 v 118 in the SRB programme; significance unclear). Thus, impacts on health were variable, and in some cases negative. The authors conclude that there is evidence of small positive health impacts, but that adverse impacts are also possible. However, they comment that the majority of UK ABI evaluations collected data solely on outputs rather than outcomes, and that therefore the scope for ABIs to tackle health inequalities is unknown. This review met six of the critical appraisal criteria; although study quality was discussed, a summary was not provided for each study, nor was the tool used to assess quality provided.

The evidence we found in this pathway maps onto the category (v), in the WHO review (outdoor environments and residential location). The risks in this category relate to poor neighbourhood quality, and perceived safety in deprived neighbourhoods as a source of inequality. The evidence from intervention studies suggests that residential mobility programmes may impact on these risks (through reducing exposure to crime). However the potential of urban regeneration programmes to impact on these risks is largely unknown, because of the lack of outcome evaluations.

### 3.2 Pathway 2: review of interventions aimed at internal housing conditions

One review included evaluations of a range of interventions designed to improve internal housing conditions (Thomson et al., 2009). This is an update of an earlier review of such interventions (Thomson et al., 2001). The authors identified 40 evaluation studies, which collected data on direct health impacts of internal housing improvements, 30 of which were relevant to this overview (4 included only child outcomes, and 6 were set in developing countries). The majority of these studies reported effects on low-income households. Interventions to improve housing conditions included warmth and energy efficiency (15 studies), rehousing and refurbishment with or without neighbourhood renewal (11 studies, 10 of UK neighbourhood renewal interventions), and rehousing from slums (4 studies). Data on general health outcomes, respiratory health, mental health and other illnesses or symptoms were extracted.

Nine studies of warmth and energy efficiency interventions reported impacts on general health outcomes; two non-randomised prospective controlled trials (one German and one UK) and two New Zealand RCTs reported significant improvements in several general health measures. Five less rigorous studies reported unclear impacts. Respiratory health impacts were reported in eleven studies of warmth interventions. The two NZ RCTs reported significant improvements in a range of measures; the remainder of the studies reported positive, unclear or conflicting impacts. Of seven studies which assessed mental health impacts, all but one reported positive effects. No consistent results were reported for the impact of warmth and energy efficiency interventions on other illnesses or symptoms.

Ten studies of rehousing or refurbishment reported findings from UK neighbourhood renewal interventions. Of six studies reporting impacts of rehousing or refurbishment on general health, three good quality studies reported small improvements, which were not statistically significant, and one study of lower quality reported a large significant increase in poor health among adults (12.3%). Three studies included respiratory health impacts, showing little evidence of improvement and reporting better outcomes for the control group on some measures. Three rigorous studies reported unclear impacts on SF-36 mental health outcomes; a further six lower quality studies reported significant positive impacts on mental health measures. One US study of refurbishment and affordable home ownership reported a significant impact on mental health, but no impacts on other outcomes. Studies that included illness or symptom impacts (3) reported no clear impacts in either direction. Four older studies reported the impacts of rehousing slum dwellers. No significant impacts were reported in any of the studies.

Overall, warmth and energy efficiency interventions seemed to have the clearest positive impacts on health. In the review the authors note that the interventions that reported the largest effects were targeted at vulnerable groups, including those with existing health conditions and the elderly. The evidence for impact of rehousing or refurbishment on physical health outcomes is as yet unclear; however, the impacts of these area-focused interventions may be diluted because area-level deprivation indices may mask considerable socio-economic heterogeneity at the individual level; thus many of those affected by area-focused interventions may not individually be in need. In most studies the intervention actually delivered to individual households varied considerably within the sample, which hampered reliable estimation of their impacts. The authors argue that there is still a need for more large-scale controlled and quasi-experimental studies, which provide more information on intervention integrity, and which permit assessment of impacts on different social groups. Study follow-up periods may be too short to detect impacts, which may take some years to manifest. The authors also comment that poor housing conditions are interlinked with other forms of deprivation; tackling internal housing conditions alone may not have major impacts on health while other determinants such as poverty and unemployment remain unchanged. All of the critical appraisal criteria were met by this review. The full quality assessment was supplied for each study, and consideration was given to the potential impact of sources of bias on the interpretation of the results.

Three of the WHO risk categories are particularly relevant to Thomson et al. (2009) (i) housing and indoor environments, (ii) fuel poverty and thermal comfort and (iii) indoor environmental exposures and overcrowding. In all of these there appears to be evidence of effective interventions, with the clearest evidence relating to category (ii). The first WHO category also includes environmental tobacco smoke in the home, which was not considered in the reviews we examined. However other evidence suggests that the evidence here is inconsistent (Edwards et al., 2009; Hyland et al., 2009).

### 3.3 Review of interventions aimed at multiple pathways

One further review included US interventions aimed at a number of pathways linking housing conditions to health (Saegert et al., 2003; the WHO literature review did not report on combinations of risks and is not discussed further here). These included rehousing and changes to: indoor equipment or furniture; respondents’ knowledge or behaviour; community norms or collective behaviour; housing policy or regulatory practices, and health practitioners’ behaviour. Seventy-two studies were included, of which 36% were interventions aimed at lead paint hazards, 35% at other safety hazards and 29% at asthma triggers or air quality hazards. Thirty-one per cent of these were aimed at low SES groups. Randomised studies (49%), and studies with a comparison group (60%), which provided data on any health outcomes, were included in the review. Forty-nine out of 72 studies reported a significant improvement in health outcomes; however, since neither the nature of the interventions, which yielded such improvements, nor the specific health outcomes, which improved are reported in the review, it is difficult to attribute any effects to specific interventions. The authors argue that policy interventions (of an unspecified nature) are relatively cost-effective, and also that ecological interventions, which target multiple levels (i.e. individuals, households, housing and neighbourhoods) are most likely to be successful. They also observe that few studies provided information on respondent SES, or on the content of interventions. They conclude that housing interventions have the potential to address health inequalities. This review met four of the seven quality appraisal criteria; the study designs were not clearly stated, the primary studies were not quality appraised, and due to the difficulty in attributing impacts to given interventions, it was deemed that the studies were not appropriately synthesised. No reference is made to assessment of study quality in this review; therefore, it is not possible to tell whether the studies were quality appraised, or whether they were considered to be of high or low quality.

## 4 Discussion

We conducted a systematic overview of housing interventions, finding five systematic reviews, which met our inclusion criteria. In total, these reviews contained 130 relevant publications evaluating the health impacts of housing interventions. The majority of the interventions included in these reviews could be considered to address health inequalities by targeting disadvantaged groups. Overall, there is evidence to suggest that interventions aimed at altering disadvantaged participants’ neighbourhood conditions by moving them to areas of lower poverty can lead to reductions in the percentage of participants reporting depression and increases in the proportion reporting good or excellent health (Acevedo-Garcia et al., 2004, Anderson et al., 2003). There is some evidence of positive impact for area effects interventions designed to improve high poverty areas, although adverse impacts can also result from these (Thomson et al., 2006). From Thomson et al. (2009), there is strong evidence that warmth and energy efficiency interventions have positive impacts on health, although the evidence on general improvements to housing conditions remains unclear. The studies synthesised by Saegert et al. (2003) led them to conclude that multiple level housing and neighbourhood interventions were most likely to be successful. However, the level of detail included in their review hampers identification of the interventions that had positive impacts.

### 4.1 Area characteristics

It appears that interventions aimed at addressing area characteristics by moving people from high to low poverty neighbourhoods can improve mental health, reduce obesity, and impact positively on some wider determinants of health, such as respondents’ experience of social disorder. It seems they have the potential to alleviate health inequalities by improving the health of disadvantaged groups. Importantly, these results lend some support to hypotheses regarding the impact of area effects on health, in that for individuals who moved to lower poverty areas, there were improvements in mental health and in obesity. However, Acevedo-Garcia et al. (2004) highlight the need for greater understanding of the specific mechanisms linking these interventions to such outcomes. Later work on the Moving to Opportunity programme has attempted to address the issue of mechanisms linking area change to health improvement; data from the nested qualitative studies suggest that moving away from an area in which there is a constant threat of random violence is the most likely explanation for the observed improvements in mental health, which may in turn lead to reductions in obesity (Kling et al., 2007), perhaps because respondents were more able to be active in a less threatening outdoors environment. It should also be noted that the MTO interventions and studies were highly complex and debate continues around certain aspects of them, notably the degree to which the findings are influenced by selection bias (Clampet-Lundquist and Massey, 2008; Sampson, 2008) and the impact of the interventions on the concentration of poverty in the sending and receiving areas (Galster, 2003; Sampson, 2008).

In relation to the policy implications of these findings, in an overview of all of the MTO studies, Kling et al. (2007) observe that it is cheaper to provide such subsidised housing vouchers than to build new public housing units, whilst also noting that the evidence on the role of area effects provided by MTO suggests that interventions to improve distressed areas may have similar impacts. Sampson (2008) suggests that area-level interventions may be more cost-effective than moving individuals to better areas, a point which has been argued by others who also point out that area-level interventions may benefit the community as a whole (Jackson et al., 2009; Leventhal and Brooks-Gunn, 2003). Thomson et al.’s (2006) review of UK ABIs investigates the impact of such interventions. Unfortunately, it seems that existing evaluations of such interventions can tell us little about their effects on health or health inequalities, although there is evidence of small positive health impacts on the residents of these deprived areas. A recent commentary by Thomson (2008) reflects on some of the obstacles to effective evaluation of such interventions, including difficulties with tracking individuals in regeneration areas, identifying suitable comparison groups or areas, and monitoring all components of these complex interventions in order to identify mechanisms leading to health changes. A further issue with area-level interventions, which focus on improving deprived areas is the difficulty of reliably assessing impacts when many of those receiving the intervention are not in the greatest need.

### 4.2 Internal housing conditions

With regards to interventions aimed at improving internal housing conditions, the review of warmth and energy efficiency interventions, housing refurbishment and relocation (Thomson et al., 2009) found strong evidence that improvements in warmth and energy efficiency have positive impacts on the health of low-income groups, particularly where these are targeted at the elderly or people with existing health conditions. Evidence on the impact of housing refurbishment and relocation remains inconclusive, although this may be due to short follow-up times, low intervention integrity or the impact of other factors such as socio-economic deprivation on intervention participants. Thus, interventions carefully targeted at those in greatest need may hold the most promise for improving health.

## 5 Research recommendations

The authors of the systematic reviews made a range of methodological recommendations. Several called for more, and more robust, prospective controlled studies (Thomson et al., 2009, Saegert et al., 2003), which collect data on intervention integrity and health impacts on different socio-economic groups (Thomson et al., 2009). The need to develop a better understanding of the mechanisms at work in housing interventions was highlighted by Acevedo-Garcia et al. (2004), who recommended qualitative methods as a means of doing so. In their earlier review of housing improvement interventions Thomson et al. (2001) also recommended qualitative methods as part of a more holistic approach to understanding pathways linking housing and health. Improved specification of intervention components and hypothesised causal pathways linking components to outcomes would lead to improved evaluations (Acevedo-Garcia, et al., 2004, Thomson et al., 2009).

In terms of substantive research topics, based on the findings of this synthesis of systematic reviews we would recommend further research on the health impacts of housing interventions aimed at housing tenure, including mixed tenure interventions and shared equity programmes, which promote affordable home ownership for low-income groups. A systematic review of mixed tenure interventions in the UK has recently been conducted and will add significantly to understanding of the evidence base on this topic (Bond et al., in press).

Methodologically, we would strongly echo calls for the greater use of mixed method studies to investigate specific mechanisms linking interventions to health outcomes; much of the evidence on mechanisms in the MTO interventions comes from the qualitative research that was conducted alongside the surveys. Indeed, health emerged as an outcome of interest during qualitative interviews conducted early in the MTO study (Acevedo-Garcia et al., 2004). This points to the increasing importance of qualitative research as a critical component of evaluation studies. We would also advocate the analysis of secondary longitudinal data to evaluate changes in structural determinants of health. An explicit equity focus should be adopted when evaluating housing interventions, so that differential impacts across different sub-groups can be assessed.

The interventions included in these reviews illustrate the difficulties inherent in disentangling the evidence on the various pathways linking health with housing and neighbourhood conditions; the studies of interventions to move people to lower poverty areas did not collect data on housing conditions, which are likely to have altered as a result of moving, and thus represent a confounding factor which may have altered the interventions’ impacts on health outcomes (Acevedo-Garcia et al., 2004). Similarly, interventions targeted at deprived areas often include measures to improve housing conditions (Thomson et al., 2006) and interventions to improve housing conditions often occur within a context of neighbourhood renewal (Thomson et al., 2009). This underscores the need for clarity in specifying intervention components and hypothesised causal pathways.

## 6 Strengths and limitations

This synthesis of systematic reviews provides an overview of current evidence on the health effects of interventions aimed at different pathways linking housing and health. By bringing together evidence from many published reports of intervention evaluations, it provides insight into what is currently known about the most promising means of improving health through altering housing conditions. There are however some limitations of the review, which should be acknowledged.

It is recognised that searching for primary studies or systematic reviews of complex social interventions, including those in the field of housing, is particularly challenging (Jackson and Waters, 2004; Ogilvie et al., 2005). Where the reviews of interest may include a wide range of interventions, study designs and outcomes, designing a search that identifies all relevant papers without generating an unmanageable number of references can be very difficult. To overcome these challenges, our search strategy was developed iteratively, piloted and revised wherever necessary to ensure maximum relevance. An experienced information scientist from the Centre for Reviews and Dissemination conducted the searches. We also hand searched appropriate journals and contacted authors. Notwithstanding these efforts, we cannot rule out the possibility that we were unable to locate some relevant reviews. Another issue is a loss of detail in the progress of information from primary studies to systematic reviews and then to systematic overviews; it is clearly not possible to provide the same level of detail in an overarching summary of reviews.

Any such synthesis of systematic reviews is also inevitably limited by the level of detail reported in the original reviews. Here we have found that reporting of intervention information is often limited, and few review authors discuss implementation issues. Reporting of quality appraisal processes and study assessment was also lacking in some cases. Where statistically significant findings were noted, it was not clear whether the review authors judged these to be clinically or socially significant. Few reviews indicated what sample sizes were available for all analyses, so it was not possible to identify whether analyses were sufficiently powered. This emphasises the importance of adhering to existing guidelines on the reporting of systematic reviews, such as those provide by PRISMA (Preferred Reporting Items for Systematic Reviews and Meta-Analyses, http://www.prisma-statement.org/). However, we feel that these issues do not detract from the utility of a systematic overview of the existing evidence base on the health impacts of housing interventions.

## 7 Conclusion

Five systematic reviews of the health effects of interventions aimed at different pathways linking housing and health were identified. Three of these reviews included studies aimed at improving area characteristics, one included studies aimed at internal housing conditions, and one included interventions aimed at a range of pathways. The lack of systematic reviews of the health impact of housing interventions aimed at altering housing tenure represents a significant gap in the systematic review evidence base on pathways linking housing and health. The findings of this systematic overview indicate that attempting to address area characteristics by moving disadvantaged people to lower poverty area appears to have some success in improving health outcomes for those who move. However, although it is cheaper than focusing investment on deprived areas, it does not help to improve conditions in these areas, thus leaving the remaining residents to contend with the existing problems. Focusing investment on deprived areas may assist all of the residents and thus be more cost-effective. However, it is difficult to gather robust evidence of impact for area-level interventions aimed at improving either area characteristics or internal housing conditions, in part because impacts may be diluted by benefiting many who are not personally disadvantaged. There are strong indications that warmth and energy efficiency interventions which are targeted at those in most need deliver at least short-term improvements in health, suggesting that interventions to improve internal housing conditions which are targeted at the most vulnerable individuals within a disadvantaged area may yield the best results. However, improved evaluation of area-level interventions may demonstrate that these also have the potential to improve health.

## Figures and Tables

**Table 1:** Summary of area effects reviews (*n*=3).

	Anderson et al. (2003)	Acevedo-Garcia et al. (2004)	Thomson et al. (2006)
**Review objective**	“to address mixed income housing programs”	“to summarize the research evidence that U.S. housing mobility policies may help improve health outcomes”	“to synthesise data on the impact on health and key socio-economic determinants of health and health inequalities reported in evaluations of national UK regeneration programmes”
**Review inclusion criteria**	Evaluation of mixed income or rental assistance intervention; before and after or no-intervention comparison; include housing hazards, neighbourhood safety, youth risk behaviour or physical/mental health outcomes.	Empirical evaluation of housing intervention; include--> ≥1 health outcome; have a comparison group	‘Evaluations that reported achievements or impacts drawing on data from at least two target areas of a national ABI programme in the UK.’
**Intervention(s)**	A. Mixed income housing developments in low SES neighbourhoods. B. Tenant based rental assistance programmes (housing vouchers allowing low SES access to more expensive areas). Participants required to remain in higher SES are for 1 year. Counselling also provided in some interventions.	Tenant based rental assistance programmes (rent subsidies in private sector requiring participants to move from high to low poverty areas). Participants required to remain in higher SES are for 1 year. Counselling also provided in some interventions.	Area-based initiatives (ABIs)—urban regeneration programmes.
**Population**	Low SES families with children	Low SES families with children	No restrictions, but most interventions aimed at deprived areas.
**Total*****N*****of included studies**	Not provided	Not provided	Not provided
**Health outcomes**	Community health; residential stability (family moves, crowded living conditions, homelessness); physical and mental health, youth behavioural problems, violence and injuries; community cohesion and civil engagement	Mental or physical health; experience of violence; substance abuse, medical care	Quality of life, well-being, health, morbidity, mortality, use of or satisfaction with local health services. Also, housing, income, education, training or employment
**Relevant primary study*****N***	12	13	3
**Study designs**	experimental, controlled and uncontrolled prospective before and after	randomised and non-randomised experimental studies	prospective cohorts, prospective repeat cross-sectional
**Database*****N***	10	8	8
**Location**	USA	USA	UK
**Synthesis method**	Narrative with median change across studies reported for some outcomes	Narrative	Narrative
**Main findings**	No studies included for intervention A.	Overall health and ‘calmness’ (1 study), distress and anxiety (1 study), depression (1 study), problem drinking and substance abuse (2 studies) improved significantly in the experimental groups. Exposure to violence decreased at follow-up (6 studies).	Impact of interventions on reported outcomes was highly variable.
	Intervention B:	Residential mobility programmes have the potential to improve health	Self-reported health: 1 before and after evaluation found deteriorations in 3 out of 4 measures of self-reported health (+ 3.8%).
	Statistically sig. improvement in 28/30 measures of crime and social disorder across 10 studies.		Mortality: 2 case study area evaluations reported improvements in standardised mortality rate: paper A=131v 114; paper B=122v 118.
	2 good quality MTO studies reported sig. improvements in 14 mental health measures and sig. improvements in self-reported health status; 11% increase in SR good/excellent health in New York, 12% increase in Boston		Authors conclude that there is ‘little evidence to demonstrate the impacts on health or socio-economic outcomes’ of ABIs, although ‘a small overall positive impact is suggested.’ However, adverse impacts of ABIs are also possible.
	1 MTO study of less good quality reported a sig. decrease of 28%-53%. in health and safety risks (e.g. rodent infestation, inadequate plumbing)		
	Tenant based rental assistance programs (2) improve household safety (crime, social disorder).		
**Quality appraisal**	1, 2, 3, 4, 5, 6	1, 2, 3, 4, 5, 6, 7	1, 2, 3, 4, 6, 7
**Quality of included studies**	Unclear	1 classed as ‘good’, 5 as ‘fair’, and 7 as ‘limited’	Not provided: authors comment overall study quality is poor

**Table 2:** Summary of housing conditions review.

	Thomson et al. (2009)
**Review objective**	“To conduct a systematic review of the health impacts of housing improvement”
**Intervention(s)**	Warmth and energy efficiency (insulation, improved central heating, improved flued heat source. Some included additional measures, e.g. light bulbs, domestic repairs 15 studies); area-based rehousing/refurbishment w/out neighbourhood renewal (11 studies); rehousing from slum conditions (4 studies).
**Review inclusion criteria**	Studies of housing improvement, which involved improving the physical attributes of the housing infrastructure’; any health or illness-related outcome measure
**Population**	All but 4 studies aimed at low-income households
**Total*****N*****of included studies**	Not provided
**Health outcomes**	General health, respiratory health, mental health, illness/symptoms
**Relevant primary study N**	30
**Study designs**	RCTs
	Prospective controlled
	Qualitative
**Database N**	42
**Location**	USA, UK, New Zealand, Europe
**Synthesis method**	Narrative with some results pooled
**Main findings**	Warmth and energy efficiency: 2 RCTs reported sig. improvements in general and respiratory health;
	2 prospective controlled studies reported sig. improvements in general health. Impacts on general and respiratory health in the remaining studies were unclear.
	6 studies reported sig. improvements in mental health.
	10 studies reported diverse and inconsistent impacts on other illness/symptoms.
	Rehousing/refurbishment: 3 better quality studies reported small insignificant improvements in general health. 1 poor study reported sig. increase in adult’s poor health (+12.3%). 3 studies found little evidence of improvement in respiratory health outcomes; in some cases, outcomes were better for the control group. 3 good quality studies found no clear impact on SF-36 mental health outcomes. A further 6 studies of lesser quality reported sig. positive impacts on a range of mental health measures. 3 studies reported mixed impacts on other illness/symptoms.
	Rehousing from slums: 4 studies conducted over 40 years ago reported no sig. impacts.
**Quality appraisal**	1, 2, 3, 4, 5, 6, 7
**Quality of included studies**	8 studies graded ‘A’, 13 graded ‘B’, and 21 graded ‘C’

**Table 3:** Summary of multiple pathways review.

	Saegert et al. (2003)
**Review objective**	“to characterize and to evaluate the success of current public health interventions related to housing”
**Review inclusion criteria**	US housing interventions to improve health; published in peer reviewed journal between January 1990 and December 2001.
**Intervention(s)**	Housing improvements: rehousing, changes in physical infrastructure, changes in indoor equipment or furniture, changes in housing policy
**Population**	USA housing residents (31% of studies related to low SES
**Total N of included studies**	Not provided
**Health outcomes**	Any health outcomes
**Relevant primary study*****N***	72
**Study designs**	44 controlled, 35 randomised studies
**Database*****N***	10
**Location**	
	USA
**Synthesis method**	
	Narrative, quantitative content analysis
**Main findings**	49/72 studies reported a sig. improvement in health outcomes (unspecified).
**Quality appraisal**	1, 2, 3, 7
**Quality of included studies**	Quality not assessed

## Box 1 Methodological quality checklist.

1. Is there a well defined question?
2. Is there a defined search strategy?
3. Are inclusion/exclusion criteria stated?
4. Are the primary study designs and number of studies clearly stated?
5. Have the primary studies been quality assessed?
6. Have the studies been appropriately synthesised?
7. Has more than one author been involved at each stage of the review process?

Source: adapted from Database of Abstracts of Reviews of Effects (DARE), (Main et al., 2008; Egan et al., 2008)

## Footnotes

Appendix A Supplementary data associated with this article can be found in the online version at doi:10.1016/j.healthplace.2010.09.011.
