# Supplementary material for: Housing and health inequalities: A synthesis of systematic reviews of interventions aimed at different pathways linking housing and health
Source: Health Place. 2011 Jan;17(1):175–84. doi: 10.1016/j.healthplace.2010.09.011 (PMC3098470; doi:10.1016/j.healthplace.2010.09.011)
Supplement: Supplementary file 1 — Supplementary material. [file mmc1.doc]

### Web Appendix 1

**Example search strategy**

**DARE**

(searched via the CAIRS interface). This is the administrative database and not available publicly

search date: 10^th^ Nov 2006

Records retrieved: 545

An additional update search was carried out on 1^st^ May 2007 and a further 177 records identified.

SEARCH[ENTER]

LIMIT D[ENTER]

S Access(2w)healthcare/til,abs,kwo[ENTER]

S Avail$(2w)healthcare/til,abs,kwo[ENTER]

S Access(2w)care/til,abs,kwo[ENTER]

S Avail$(2w)care/til,abs,kwo[ENTER]

S Care(w)seeking(w)behaviour/til,abs,kwo[ENTER]

S Care(w)seeking(w)behavior/til,abs,kwo[ENTER]

S social(2w)depriv$/til,abs,kwo[ENTER]

S social(2w)disadvantage$/til,abs,kwo[ENTER]

S Educational(w)achievement/til,abs,kwo[ENTER]

S Educational(w)status/til,abs,kwo[ENTER]

S equity/til,abs,kwo[ENTER]

S Financial(w)difficult$/til,abs,kwo[ENTER]

S Financial(w)problem$/til,abs,kwo[ENTER]

S Health(w)difference$/til,abs,kwo[ENTER]

S Health(2w)disparit$/til,abs,kwo[ENTER]

S Health(w)equit$/til,abs,kwo[ENTER]

S Health(2w)inequalit$/til,abs,kwo[ENTER]

S Health(2w)inequit$/til,abs,kwo[ENTER]

S Health(w)service$(w)access$/til,abs,kwo[ENTER]

S Health(2w)variation$/til,abs,kwo[ENTER]

S Income/til,abs,kwo[ENTER]

S Income(w)difference$/til,abs,kwo[ENTER]

S Indigent/til,abs,kwo[ENTER]

S Inequity/til,abs,kwo[ENTER]

S Insurance(2w)health/til,abs,kwo[ENTER]

S Insurance(2w)status/til,abs,kwo[ENTER]

S jobless/til,abs,kwo[ENTER]

S job(w)insecurity/til,abs,kwo[ENTER]

S Low(w)income/til,abs,kwo[ENTER]

S Marginalised/til,abs,kwo[ENTER]

S Marginalized/til,abs,kwo[ENTER]

S Material(w)depriv$/til,abs,kwo[ENTER]

S Medical$(w)indigen$/til,abs,kwo[ENTER]

S Medically(w)uninsured/til,abs,kwo[ENTER]

S medicare/til,abs,kwo[ENTER]

S Multipl$(w)depriv$/til,abs,kwo[ENTER]

S Occupational(w)status/til,abs,kwo[ENTER]

S Poverty/til,abs,kwo[ENTER]

S Psychosocial(w)depriv$/til,abs,kwo[ENTER]

S Rural(w)health/til,abs,kwo[ENTER]

S SES/til,abs,kwo[ENTER]

S Social(w)capital/til,abs,kwo[ENTER]

S Social(w)class/til,abs,kwo[ENTER]

S Social(w)condition$/til,abs,kwo[ENTER]

S Social(w)depriv$/til,abs,kwo[ENTER]

S Social(w)difference$/til,abs,kwo[ENTER]

S Social(w)disparit$/til,abs,kwo[ENTER]

S Social(w)environment/til,abs,kwo[ENTER]

S Social(w)exclusion/til,abs,kwo[ENTER]

S Social(w)factor$/til,abs,kwo[ENTER]

S Social(w)gradient$/til,abs,kwo[ENTER]

S Social(w)inclusion/til,abs,kwo[ENTER]

S Social(w)inequalit$/til,abs,kwo[ENTER]

S Social(w)inequit$/til,abs,kwo[ENTER]

S Social$(w)isolat$/til,abs,kwo[ENTER]

S Social(w)justice/til,abs,kwo[ENTER]

S Social(w)position/til,abs,kwo[ENTER]

S Social(w)security/til,abs,kwo[ENTER]

S Social(w)variation$/til,abs,kwo[ENTER]

S Social(w)welfare/til,abs,kwo[ENTER]

S Social$(w)exclu$/til,abs,kwo[ENTER]

S Socio(w)economic(w)attribution$/til,abs,kwo[ENTER]

S Socio(w)economic(w)circumstance$/til,abs,kwo[ENTER]

S Socio(w)economic(w)factor$/til,abs,kwo[ENTER]

S Socio(w)economic(w)gradient$/til,abs,kwo[ENTER]

S Socio(w)economic(w)health(w)difference$/til,abs,kwo[ENTER]

S Socio(w)economic(w)position/til,abs,kwo[ENTER]

S Socio(w)economic(w)status/til,abs,kwo[ENTER]

S Socio(w)economic(w)variable$/til,abs,kwo[ENTER]

S Socioeconomic(w)attribution$/til,abs,kwo[ENTER]

S Socioeconomic(w)circumstance$/til,abs,kwo[ENTER]

S Socioeconomic(w)factor$/til,abs,kwo[ENTER]

S Socioeconomic(w)gradient$/til,abs,kwo[ENTER]

S Socioeconomic(w)health(w)difference$/til,abs,kwo[ENTER]

S Socioeconomic(w)position/til,abs,kwo[ENTER]

S Socioeconomic(w)status/til,abs,kwo[ENTER]

S Socioeconomic(w)variable$/til,abs,kwo[ENTER]

S Standard(2w)living/til,abs,kwo[ENTER]

S State(w)benefits/til,abs,kwo[ENTER]

S Uncompensated(w)care/til,abs,kwo[ENTER]

S Underinsur$(2w)health/til,abs,kwo[ENTER]

S Underprivilege$/til,abs,kwo[ENTER]

S Unemployed/til,abs,kwo[ENTER]

S unemployment/til,abs,kwo[ENTER]

S Uninsur$(2w)health/til,abs,kwo[ENTER]

S Urban(w)health/til,abs,kwo[ENTER]

S Vulnerable(w)population/til,abs,kwo[ENTER]

s vulnerable(w)group$/til,abs,kwo[ENTER]

s vulnerable(w)communit$/til,abs,kwo[ENTER]

s vulnerable(w)people/til,abs,kwo[ENTER]

s vulnerable(W)person$/til,abs,kwo[ENTER]

S Welfare/til,abs,kwo[ENTER]

S workless/til,abs,kwo[ENTER]

S worklessness/til,abs,kwo[ENTER]

S S1 OR S2 OR S3 OR S4 OR S5 OR S6 OR S7 OR S8 OR S9[ENTER]

S S10 OR S11 OR S12 OR S13 OR S14 OR S15 OR S16 OR S17 OR S18 OR S19[ENTER]

S S20 OR S21 OR S22 OR S23 OR S24 OR S25 OR S26 OR S27 OR S28 OR S29[ENTER]

S S30 OR S31 OR S32 OR S33 OR S34 OR S35 OR S36 ORS 37 OR S38 OR S39[ENTER]

S S40 OR S41 OR S42 OR S43 OR S44 OR S45 OR S46 OR S47 OR S48 OR S49[ENTER]

S S50 OR S51 OR S52 OR S53 OR S54 OR S55 OR S56 OR S57 OR S58 OR S59[ENTER]

S S60 OR S61 OR S62 OR S63 OR S64 OR S65 OR S66 OR S67 OR S68 OR S69[ENTER]

S S70 OR S71 OR S72 OR S73 OR S74 OR S75 OR S76 OR S77 OR S78 OR S79[ENTER]

S S80 OR S81 OR S82 OR S83 OR S84 OR S85 OR S86 OR S87 OR S88 OR S89[ENTER]

S S90 OR S91 OR S92 OR S93 OR S94[ENTER]

S S95 OR S96 OR S97 OR S98 OR S99 OR S100 OR S101 OR S102 OR S103 OR S104[ENTER]
